# Connexin43 and connexin50 channels exhibit different permeability to the second messenger inositol triphosphate

**DOI:** 10.1038/s41598-020-65761-z

**Published:** 2020-05-26

**Authors:** Virginijus Valiunas, Thomas W. White

**Affiliations:** The Department of Physiology and Biophysics, Stony Brook University School of Medicine, Stony Brook, NY 11794 USA

**Keywords:** Permeation and transport, Ion channel signalling

## Abstract

Gap junction channels made of different connexins have distinct permeability to second messengers, which could affect many cell processes, including lens epithelial cell division. Here, we have compared the permeability of IP_3_ and Ca^2+^ through channels made from two connexins, Cx43 and Cx50, that are highly expressed in vertebrate lens epithelial cells. Solute transfer was measured while simultaneously monitoring junctional conductance via dual whole-cell/perforated patch clamp. HeLa cells expressing Cx43 or Cx50 were loaded with Fluo-8, and IP_3_ or Ca^2+^ were delivered via patch pipette to one cell of a pair, or to a monolayer while fluorescence intensity changes were recorded. Cx43 channels were permeable to IP_3_ and Ca^2+^. Conversely, Cx50 channels were impermeable to IP_3_, while exhibiting high permeation of Ca^2+^. Reduced Cx50 permeability to IP_3_ could play a role in regulating cell division and homeostasis in the lens.

## Introduction

The proper growth and development of vertebrate tissues relies upon chemical communication between adjacent cells. This can result from the activation of cytoplasmic signal transduction cascades by extracellular growth factors to generate second messengers, or can directly occur between adjacent cells through the gap junction channels that link their cytoplasm. Both gap junctional communication and growth factor signaling pathways have been shown to play critical roles in the development and growth of the lens^1–9^. However, few studies have examined if differential permeability to second messenger molecules by the connexin channels expressed in the lens epithelium, Cx43 and Cx50, could play a role in the specificity of cell-to-cell communication during eye development^10–12^.

Initial reports assumed that gap junction channels would be relatively non-selective^13,14^, however connexin-dependent differences in ion and small molecule permeability were subsequently identified^15–18^. Further studies extended connexin permeation differences to second messenger molecules such as IP_3_ and cAMP^10,19–23^. Currently, it is thought that each type of connexin channel has functionally distinct ionic conductance and small molecule permeability^19,20,24–26^. The importance of permeation differences between connexin channel types *in vivo* has been suggested by mouse genetic studies showing that loss of one connexin often cannot be compensated for by replacement with another^27–32^.

Animal models with genetic manipulations of Cx43 and Cx50 have consistently supported this idea^28,33–37^, and the lens is an excellent model system to explore differential permeability of gap junctions to second messengers. There is a well-established literature on the pathological effects resulting from genetically manipulating the lens connexins *in vivo*^38–41^, that can be contrasted with changes in second messenger permeation observed *in vitro*. Here, we have used a combined patch clamp electrophysiological and fluorescent imaging approach to compare the permeation of IP_3_ and Ca^2+^ through Cx43 and Cx50 channels *in vitro*. We found that Cx43 channels were permeable to both IP_3_ and Ca^2+^, whereas channels composed of Cx50 showed undetectable permeability to IP_3_, but high permeability to Ca^2+^ ions that was comparable to that of Cx43.

## Results

### Cx43 channels are permeable to IP_3_

**Figure 1:**
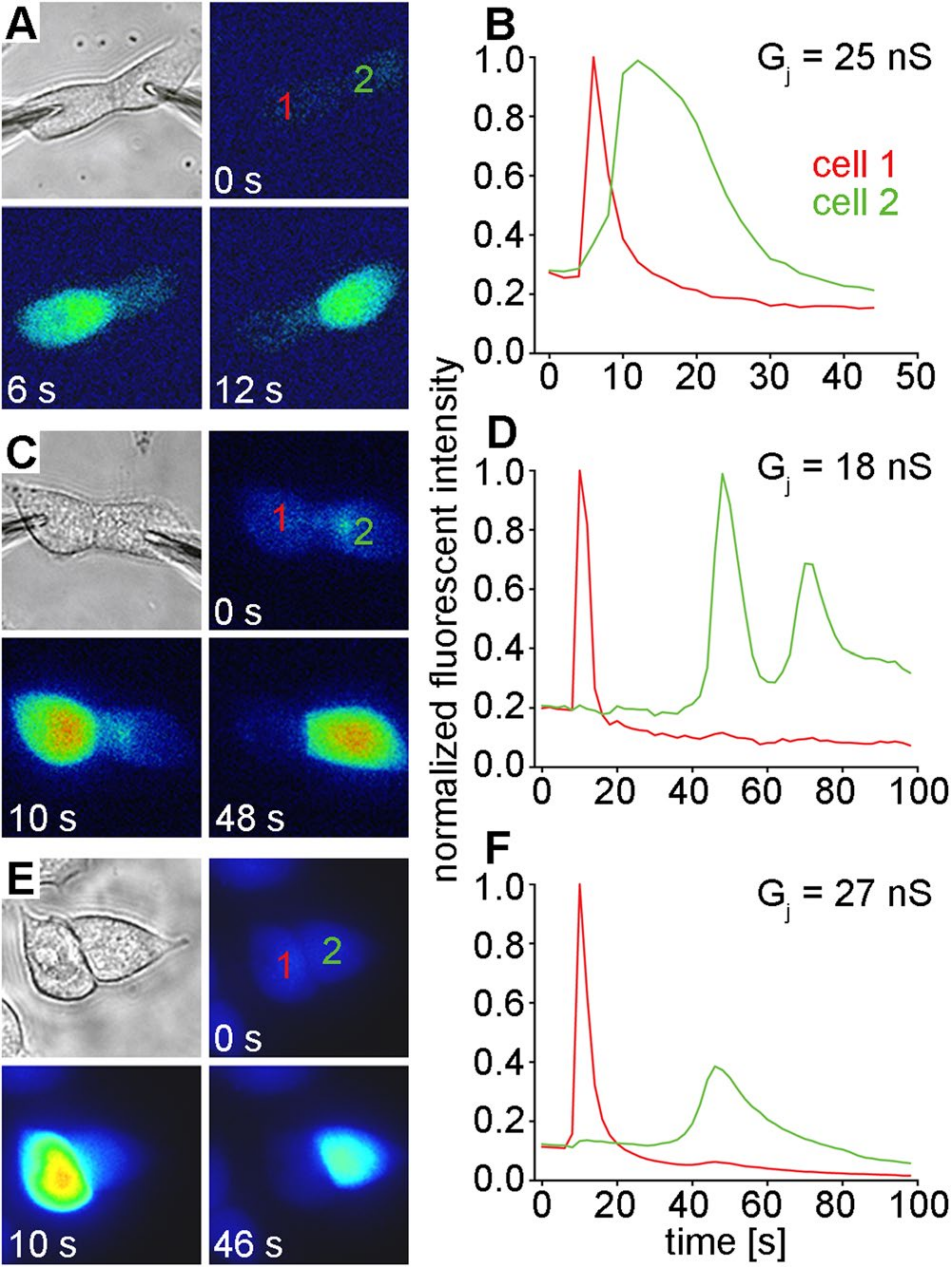
Cx43 channels are permeable to IP_3_. Three different Cx43 cell pairs are shown (**A,C,E**) that all showed increased Ca^2+^ fluorescence in cell 1 (red lines) when IP_3_ was introduced, and 10 to 40 seconds later a response in cell 2 (green lines) after it had permeated through Cx43 channels (**B,D,F**). In the pairs shown in A-D, 500 µM IP_3_ was in the pipette. In the pair shown in E-F, 250 µM IP_3_ was in the pipette. The mean gap junctional conductance (± SD) for the three Cx43 cell pairs shown was 23 ± 4.7 nS.

Permeability of connexin channels to IP_3_ has been documented in a number of experimental systems, most frequently for channels composed of Cx26^21–23,42–44^. Many of these studies utilized IP_3_ mediated ER calcium release^45,46^ and Ca^2+^ sensitive fluorescent dyes to detect IP_3_ permeation through gap junction channels. We analyzed the IP_3_ permeability of Cx43 channels using cell pairs loaded with the Ca^2+^ binding dye Fluo-8 (Fig. 1). In all examples shown, cell 1 was patched in whole cell mode and IP_3_ was added to the pipette solution. Cell 2 was patched in the perforated patch mode to simultaneously measure gap junctional conductance while imaging cell fluorescence (for experimental details see Materials and Methods). Cell fluorescence was initially monitored for 15–40 seconds to establish a baseline, then the whole cell mode with the pipette attached to cell 1 was established to release 500 µM IP_3_ into the cytoplasm. A rapid spike in fluorescent intensity was observed in cell 1 (red traces), which rapidly attenuated due to the presence of 10 mM EGTA in the pipette solution. For every cell pair expressing Cx43 that we tested (n = 16), cell 2 showed a spike in fluorescent intensity (green traces) between 10 and 40 seconds after IP_3_ was delivered to cell 1, indicating permeation through Cx43 gap junction channels. In rare cell pairs (Fig. 1C,D), the fluorescent response in the recipient cell showed Ca^2+^ oscillation, although we cannot conclusively explain why this occurred. In these cases, the first major response was counted as the positive result. In the third cell pair shown (Fig. 1E,F), the concentration of IP_3_ in the pipette solution was reduced to 250 µM. There were no statistically significant differences in the mean times (± SD) between the fluorescent peaks observed in cell 1 and cell 2 when the IP_3_ concentration in the pipette solution was varied (16.2 ± 10.6 seconds, n = 9, for 500 μM versus 18.9 ± 11.3 seconds, n = 7 for 250 μM, p = 0.64, student’s t-test). The mean gap junctional conductance (± SD) for all of the Cx43 cell pairs investigated as shown in Fig. 1 was 21 ± 9.7 nS (n = 16).

### IP_3_ cannot permeate Cx50 channels

**Figure 2:**
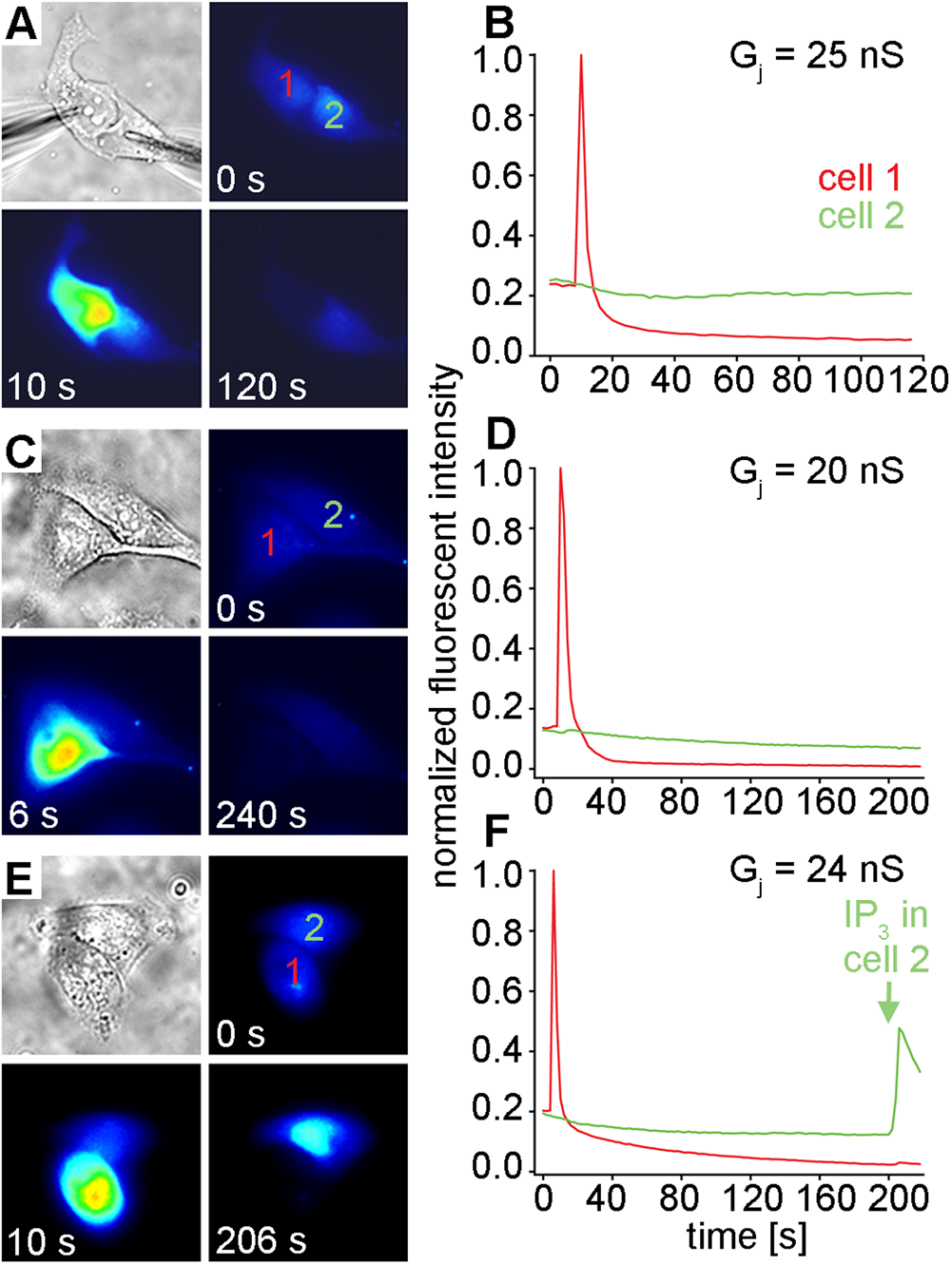
IP_3_ does not permeate channels made of Cx50. Three different Cx50 cell pairs (**A,C,E**) all showed a rapid spike in Ca^2+^ fluorescent intensity in cell 1 (red lines) when IP_3_ was introduced. In contrast to Cx43, cell 2 (green lines) never showed any spike in fluorescent intensity (**B,D,E**) for up to 220 seconds following IP_3_ delivery to cell 1, indicating no permeation through Cx50 channels. When 500 µM IP_3_ was directly introduced into cell 2 after monitoring fluorescence for 200 seconds following IP_3_ release into cell 1 (**E,F**), a rapid spike in fluorescent intensity was observed in cell 2 within seconds. The mean gap junctional conductance (± SD) for the three Cx50 cell pairs shown was 23 ± 2.6 nS.

When we analyzed the IP_3_ permeability of Cx50 channels using the same method (Fig. 2), a rapid spike in fluorescent intensity was observed in cell 1 (red traces), which rapidly attenuated, just as we had observed for Cx43. In contrast to Cx43, for every cell pair expressing Cx50 that we tested (n = 18), cell 2 never showed any spike in fluorescent intensity (green traces) for up to 220 seconds following IP_3_ delivery to cell 1, indicating no detectable permeation of this second messenger through Cx50 gap junction channels. In the third cell pair shown (Fig. 2E,F), we directly introduced 500 µM IP_3_ into cell 2 after monitoring fluorescence for 200 seconds following IP_3_ release into cell 1. We observed a rapid spike in fluorescent intensity in cell 2 within seconds, demonstrating that cell 2 was capable of responding to IP_3_, and that the lack of response was due to the absence of its permeation through Cx50 channels. The mean gap junctional conductance (± SD) for all of the Cx50 cell pairs tested as shown in Fig. 2 was 21 ± 11.6 nS (n = 18), the same magnitude of coupling provided by Cx43 channels in the data presented in Fig. 1.

### Cx43 and Cx50 channels are permeable to Ca^2+^

**Figure 3:**
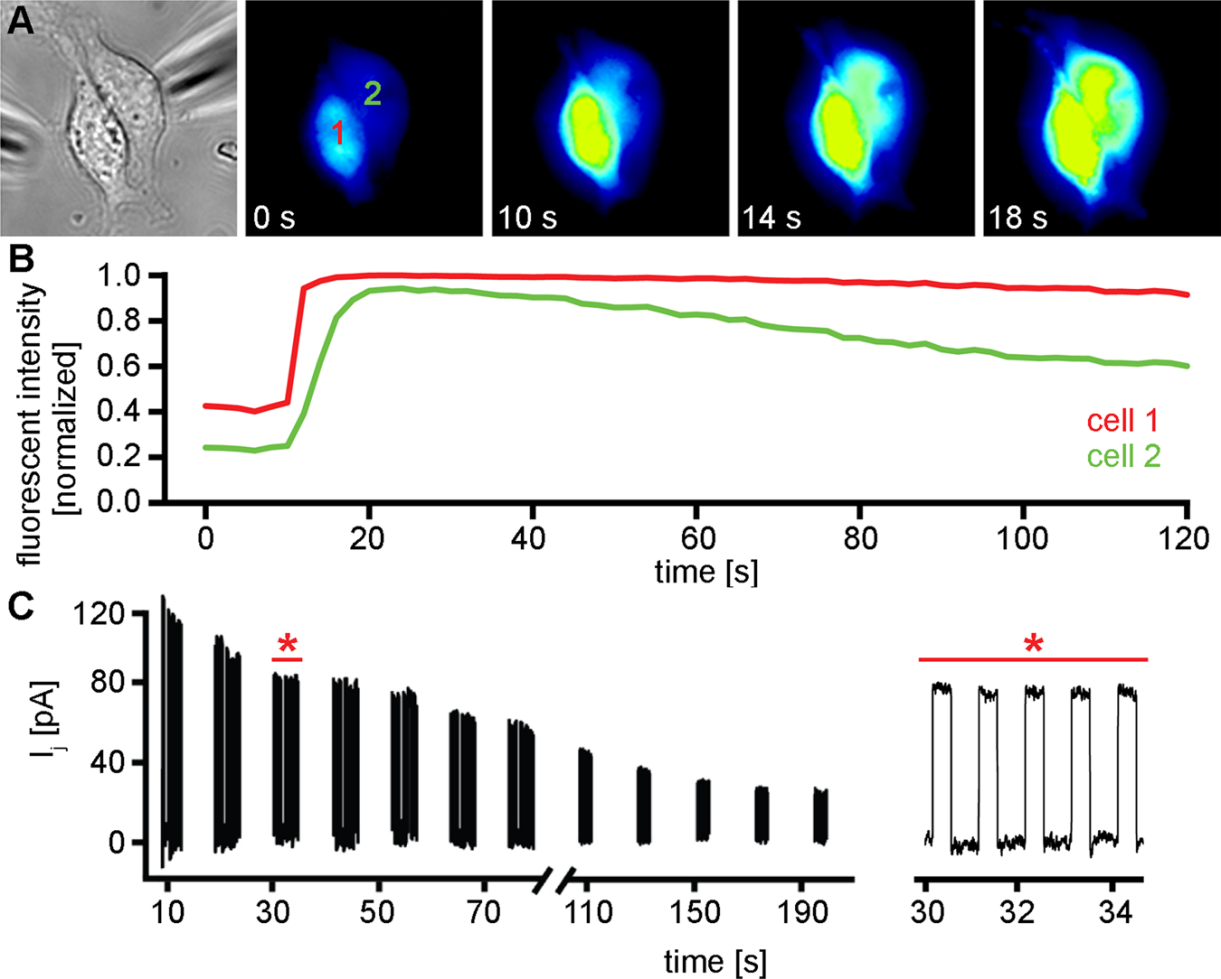
Cx50 Channels are permeable to Ca^2+^ ions. A cell pair expressing Cx50 (**A**), showed a strong fluorescent response in cell 1 (**B**, red line), when the pipette attached to cell 1 was opened to release 2 mM Ca^2+^ into the cytoplasm. Cell 2 showed a rise in fluorescent intensity (**B**, green line) that reached a peak within 10 seconds after Ca^2+^ was delivered to cell 1. Simultaneous measurement of gap junctional currents (**C**) showed that Cx50 was gated by Ca^2+^ on a much slower time scale than the solute permeation. A less than 50% decline in the Cx50 junctional current was seen 60 seconds after 2 mM Ca^2+^ was delivered to cell 1, which increased to a 77% decline in coupling at 180 seconds. An expanded portion of the record (red asterisk) showed that junctional currents remained stable for many seconds after peak Ca^2+^transfer had occurred.

Connexin channels have been shown to be both permeable to and gated by Ca^2+^^44,47–50^. For the lens connexins, physiologically relevant changes in the levels of cytoplasmic calcium have been reported to markedly reduce gap junctional coupling^51,52^. To ensure that differential effects of Ca^2+^ on conductance of the two channel types did not cause the striking disparity in IP_3_ permeability observed between Cx43 and Cx50, we examined the Ca^2+^ permeability and gating of Cx50 using cell pairs loaded with Fluo-8 (Fig. 3). Cell 1 was patched in whole cell mode and 2 mM Ca^2+^ was added to the pipette solution. Cell 2 was patched in whole cell mode to simultaneously measure gap junctional conductance while continuously imaging Fluo-8 fluorescence. Fluorescence was initially monitored for 10–30 seconds to establish a baseline, then the patch was ruptured under the pipette attached to cell 1 to establish the whole cell mode and to release Ca^2+^ into the cytoplasm. A rapid rise in fluorescent intensity was observed in cell 1 (red trace), which persisted due to the omission of 10 mM EGTA from the pipette solution. For every cell pair expressing Cx50 that we tested (n = 4), cell 2 showed a rise in fluorescent intensity (green traces) between ~ 5 and 20 seconds after Ca^2+^ was delivered to cell 1, indicating permeation through Cx50 gap junction channels. The simultaneous measurement of gap junctional conductance showed that Cx50 was also gated by Ca^2+^, as was previously documented^53^, although on a much slower timescale than the solute permeation. A ~50% decline in the Cx50 junctional current was observed 60 seconds after 2 mM Ca^2+^ was delivered to cell 1, which increased to a 77% decline in coupling at 180 seconds. The mean gap junctional conductance (± SD) for all of the Cx50 cell pairs used in calcium permeability studies was 14 ± 5.1 nS.

**Figure 4:**
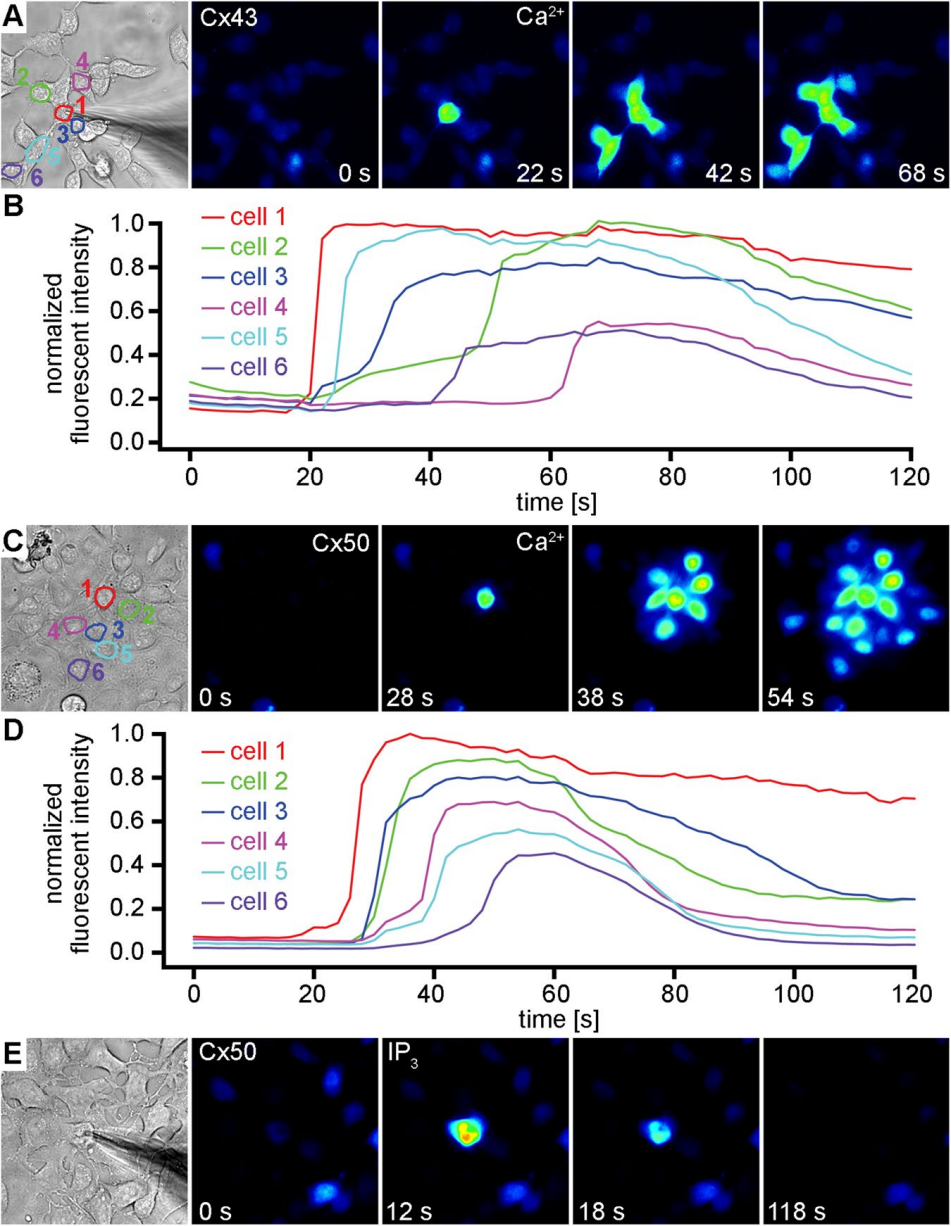
Ca^2+^ and IP_3_ permeation in monolayer cultures of Cx43 and Cx50 expressing cells. After recording background fluorescence for ~20 seconds, 2 mM Ca^2+^ was released into a single cell in a cluster of Cx43 expressing cells loaded with Fluo-8 (**A**). Fluorescence rapidly increased in the patched cell, followed by an increase in 5 more distal cells (**B**) within 60 seconds. Similar (**C–D**) results were obtained when 2 mM Ca^2+^ was introduced into the cytoplasm of a single cell within a cluster of Cx50 expressing cells. In contrast, delivery of 500 µM IP_3_ into the cytoplasm of a single cell within a cluster of Cx50 expressing cells (**E**) resulted in a rapid peak of fluorescent intensity in the injected cell, with no evidence of IP_3_ permeation to any of the adjacent cells.

We also found that Cx43 channels were highly permeable to Ca^2+^, although we used a more simplified assay (Fig. 4A,B, n = 5)). In these experiments, Cx43 expressing cells were loaded with Fluo-8 and a single cell in a multicellular cluster was patched in the whole cell mode with 2 mM Ca^2+^ added to the pipette solution while fluorescence was continuously monitored. Fluo-8 fluorescence rapidly increased in the patched cell, followed by a fluorescence increase in more distal cells in contact with the patched cell within one minute. Similar results were obtained with Cx50 expressing cells, when 2 mM Ca^2+^ was introduced into the cytoplasm of a single cell within a large cluster of cells (Fig. 4C,D, n = 3). For these Ca^2+^ permeability studies, 10 mM EGTA was omitted from the pipette solution. In contrast, delivery of 500 µM IP_3_ into the cytoplasm of a single cell within a large cluster of Cx50 expressing cells resulted in a rapid peak of fluorescent intensity in the injected cell, with no evidence of IP_3_ permeation to adjacent cells (Fig. 4E, n = 3). For these IP_3_ permeability studies, 10 mM EGTA was present in the pipette solution. These results confirmed that while both Cx43 and Cx50 channels showed a high permeability to Ca^2+^, only Cx43 displayed detectable permeability to the second messenger IP_3_.

## Discussion

We have contrasted IP_3_ and Ca^2+^ permeability through gap junction channels formed from the lens connexins, Cx43 and Cx50. Cx50 showed greatly reduced IP_3_ permeability compared to Cx43, while both connexins were readily permeable to Ca^2+^. Differences in the permeation of these second messengers through Cx43 and Cx50 channels could influence the development of cataract in the lens. Calcium has long been known to play a significant role in cataract formation^54,55^ and altered Ca^2+^ signaling in lens epithelial cells has been implicated in cataract progression^56^. In addition, the lens possesses an array of G-protein coupled receptors that facilitate the release of intracellular calcium through the generation of IP_3_^57–59^ and gap junction mediated Ca^2+^ signaling has been documented in primary cultures of lens epithelial cells^60^. Since the intercellular movement of IP_3_ appears to be more important for cell-to-cell propagation of Ca^2+^ signals^43,61^ (supplementary Fig. S1), the profoundly different permeability of Cx43 and Cx50 to IP_3_ could be relevant in cataract progression in the lens.

Differential permeability of IP_3_ through Cx43 and Cx50 channels could also impact lens cell proliferation and growth during development. Cx50 knockout decreased epithelial cell division during the first post-natal week, resulting in a significant reduction of lens growth^7,34,62^. In contrast, deletion of Cx43 did not reduce lens growth^36,63^, suggesting that a specific functional difference between Cx43 and Cx50 was required for normal postnatal epithelial mitosis and lens growth to occur. IP_3_ and Ca^2+^ act synergistically to influence cell division^64–66^, including in cultured human lens epithelial cells^67^. Elevated IP_3_ levels lead to Ca^2+^ release from the endoplasmic reticulum^68^, potentially linking second messenger generation following receptor activation to permeability properties of connexin channels. If restricted IP_3_ permeability through connexin channels were important for normal epithelial cell division during postnatal development, then one would predict that Cx43 could not compensate for loss of Cx50 in the lens epithelium, as it exhibits much greater permeability to IP_3_.

Recently, the structure of Cx50 has been resolved by cryo-electron microscopy at a resolution near the atomic level (~3.4 Å)^69^. Although there is no equivalent structure of Cx43 at this resolution, the availability of a Cx50 structure may allow approaches such as comparative all-atom MD simulations to probe isoform-specific differences in perm-selectivity to second messengers like IP_3_. Cx26 has been shown to be permeable to IP_3_^22,43^, and there is also an atomic level structure for this connexin^70^, so this approach would not absolutely require an atomic level structure of Cx43. The documentation of profound differences in the permeation of connexin channels to biologically relevant second messengers is an important step in understanding the need for connexin diversity to maintain homeostasis in a variety of biological systems^29,71^. In combination with increasing information about connexin channel atomic structure, the molecular basis for these permeability differences may finally be elucidated in future studies.

## Materials and Methods

### Cell lines

HeLa cells that had been previously stably transfected with rat Cx43, or human Cx50^17,72^ were cultured and plated as described^10^. Briefly, cells were grown in DMEM (Gibco/Thermo Fisher Scientific, Waltham, MA), supplemented with 10% FCS (Hyclone/ Thermo Fisher Scientific, Waltham, MA), 100 μg/mL streptomycin, and 100 U/mL penicillin (Gibco) and were passaged weekly, diluted 1:10, and kept at 37 °C in a CO_2_ incubator (5% CO_2_ / 95% ambient air). Permeability measurements were carried out on cell pairs plated on glass coverslips at low density.

### Patch clamp electrophysiology

A combined whole-cell/perforated patch dual voltage-clamp method was used to measure gap junctional conductance between cell pairs^17,73^. Coverslips with cells expressing Cx43, or Cx50, were transferred to a recording chamber on an inverted microscope with a fluorescence imaging system. Cells were perfused with a solution containing (in mM) NaCl, 150; KCl, 10; CaCl_2_, 2; HEPES, 5 (pH 7.4); glucose, 5; CsCl, 2; and BaCl_2_, 2. Patch clamp electrodes were filled with a solution containing (in mM) K^+^ aspartate^-^, 120; NaCl, 10; MgATP, 3; HEPES, 5 (pH 7.2); EGTA, 10. For electrodes used in a perforated patch configuration, the solution was supplemented with 30–50 μM β-escin^74^. Patch clamp electrodes were pulled from glass capillaries (Harvard Apparatus, Holliston, MA) with a horizontal puller (DMZ-Universal, Zeitz-Instrumente, Martinsried, Germany). The measured resistance of the electrodes was 2–5 MΩ. Gap junctional conductance was measured throughout each experiment, beginning when both cells in the pair had been successfully patched.

### IP_3_ permeability studies

**Figure 5:**
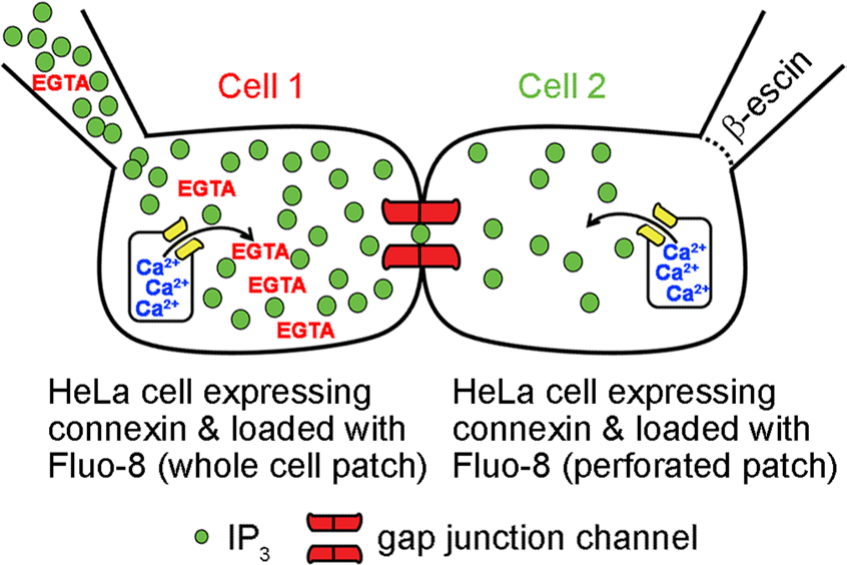
An assay for intercellular transfer of IP_3_ through gap junction channels. Cell pairs are cultured where both cells express the connexin to be tested, and are loaded with the Ca^2+^ indicator Fluo-8. 500 µM IP_3_ is delivered via patch pipette into cell 1 in whole cell mode, and Fluo-8 fluorescence is continuously recorded in both cells using a digital camera. When IP_3_ transfers to cell 2 through connexin channels, the liberation of Ca^2+^ from ER stores can be detected. Cell 2 is patched in the perforated mode to simultaneously record junctional conductance.

We used IP_3_ mediated ER calcium release^45,46^ and Ca^2+^ sensitive fluorescent dyes as an assay for IP_3_ permeation through gap junction channels (Fig. 5). The detection of intercellular IP_3_ transfer was accomplished by culturing cell pairs where both cells expressed the connexin to be tested, and were pre-loaded with the calcium indicator dye Fluo-8 (5 μM, AAT Bioquest, Sunnyvale, CA) according the manufacturer’s protocol. IP_3_ transfer was monitored by continuously recording fluorescence in both cells using a digital CCD-camera (HRm Axiocam, Carl Zeiss, Thornwood, NY), while simultaneously measuring junctional conductance between cells 1 & 2 after the delivery of 250 or 500 µM IP_3_ to cell 1 through the patch pipette. An outline of each cell, and an equally sized area of background in an adjacent area without cells, were manually drawn in the images using AxioVision Software (Carl Zeiss, White Plains, NY). Fluorescent intensities for recipient and source cells were corrected by subtracting the background intensity, normalized to the peak fluorescent intensity in cell 1 and plotted versus time. To ensure that we monitored the passage of IP_3_, and not endogenous free Ca^2+^, which would also be liberated in the source cell, the pipette solution contained 10 mM EGTA to chelate the released Ca^2+^ in cell 1, and prevent its passage to cell 2 through gap junction channels. However, previous reports^43,61,75,76^ and additional control experiments (Supplementary Figure S1) suggested that the cell-to-cell transfer of endogenous Ca^2+^ was likely too minimal to confound our ability to detect IP_3_ transfer. Cell 2 was recorded in the perforated patch mode to prevent IP_3_ from diffusing into the recipient cell pipette. To prevent endogenous IP_3_ synthesis or degradation within the source cell, 3.5 mM diphosphoglyceric acid (a competitive inhibitor of IP_3_ phosphomonoesterases) and 20 µM IP_3_-kinase inhibitor (MilliporeSigma, Burlington, MA) were included in the pipette solution^42,77^.

### Ca^2+^ permeability studies

Connexin expressing cell pairs loaded with Fluo-8 were also used to directly monitor Ca^2+^ permeability through gap junction channels. The detection of intercellular Ca^2+^ transfer was accomplished by culturing cell pairs where both cells expressed the connexin to be tested, and were pre-loaded with Fluo-8. Ca^2+^ transfer was monitored by continuously recording fluorescence in both cells, while simultaneously measuring junctional conductance between cells 1 & 2 after the delivery of 2 mM Ca^2+^ to cell 1. In some experiments, a single cell in a multicellular cluster of connexin cells was patched in the whole cell mode with 2 mM Ca^2+^ added to the pipette solution while Fluo-8 fluorescence was continuously monitored in the entire field of adjacent cells. For these studies, 10 mM EGTA was omitted from the pipette solution.

## Supplementary information

Supplementary Information.
