# Supplementary material for: Connexin43 and connexin50 channels exhibit different permeability to the second messenger inositol triphosphate
Source: Sci Rep. 2020 May 26;10:8744. doi: 10.1038/s41598-020-65761-z (PMC7251084; doi:10.1038/s41598-020-65761-z)
Supplement: Supplementary file 1 — Supplementary Information. [file 41598_2020_65761_MOESM1_ESM.pdf]

Supplementary Information for the article:

Connexin43 and connexin50 channels exhibit different permeability to the second messenger inositol triphosphate. Virginijus Valiunas, and Thomas W. White

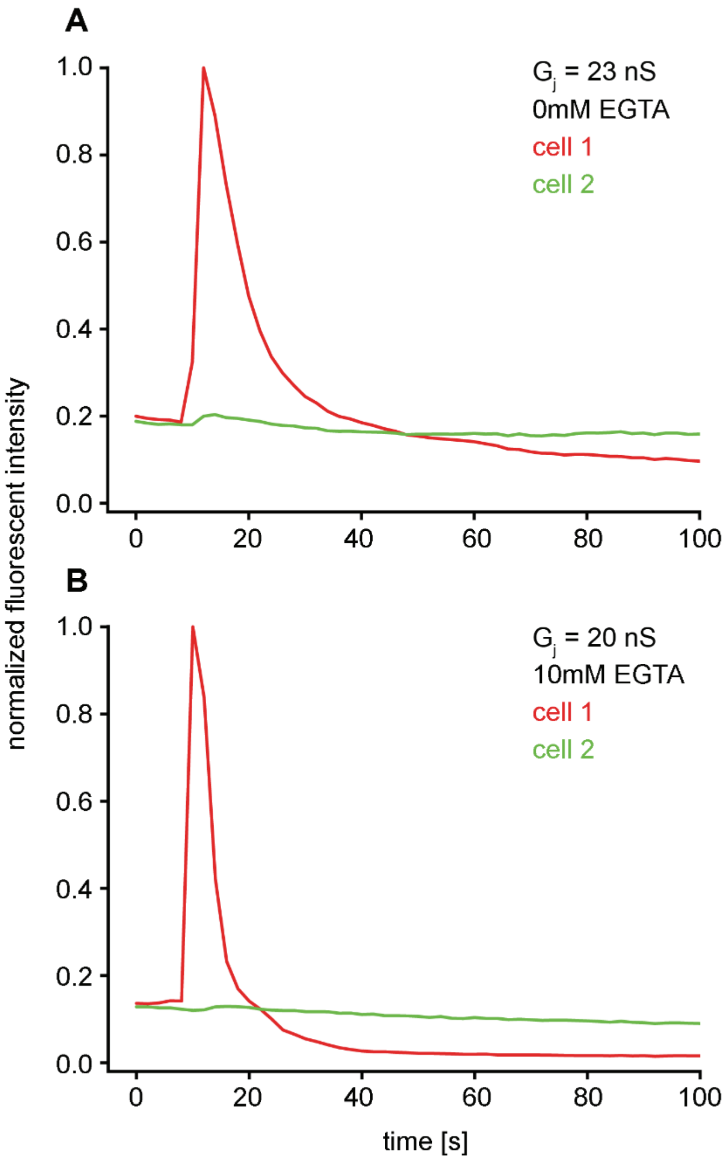

Supplementary Figure S1. The effect of EGTA concentration in the whole cell patch pipette. Examples of two different Cx50 expressing cell pairs where the pipette solution used to deliver IP_3 contained either 0mM (A), or 10mM (B) of the calcium chelator EGTA. Both cell pairs showed a rapid spike in Ca^{2+} fluorescent intensity in cell 1 (red lines) when IP_3 was introduced, but the peak was much broader in the absence of EGTA (A, 38 seconds duration) than in the presence of 10mM EGTA (B, 10 seconds duration), showing that EGTA is an effective chelator of cytoplasmic Ca^{2+}. In both cases, cell 2 (green lines) showed negligible increases in fluorescent intensity following IP_3 delivery to cell 1, indicating that the transient levels of cytoplasmic Ca^{2+} liberated by IP_3 were too low, or too rapidly cleared by endogenous Ca^{2+} pumps for their passage through Cx50 channels to be detected. In either case, the data suggest that the cell-to-cell transfer of endogenous Ca^{2+} did not interfere with our ability to detect IP_3 transfer. The total magnitude of gap junctional conductance in both of the cell pairs was similar.
